# Diagnosing Emergent Heterotopic Pregnancy via Point-of-Care Ultrasound: A Case Report

**DOI:** 10.7759/cureus.43663

**Published:** 2023-08-17

**Authors:** William N Doyle, Chelsea C Giagni, Kevin Roth, Alexandra Amaducci

**Affiliations:** 1 Department of Emergency and Hospital Medicine, Lehigh Valley Health Network / University of South Florida, Morsani College of Medicine, Bethlehem, USA; 2 Department of Obstetrics and Gynecology, Lehigh Valley Health Network / University of South Florida, Morsani College of Medicine, Bethlehem, USA

**Keywords:** maternal morbidity, diagnostic medicine, ovulation induction, heterotopic pregnancy, pocus

## Abstract

Patients undergoing assisted reproductive treatments are at a much higher risk for developing heterotopic pregnancy, a rare complication marked by concurrent intrauterine and ectopic pregnancies. Ruptured ectopic pregnancies are one of the leading causes of pregnancy-related mortality. We report the case of a 31-year-old woman undergoing ovulation induction that presented to the emergency department (ED) with worsening abdominal pain. Point-of-care ultrasound (POCUS) performed in the ED identified a heterotopic pregnancy in which the ectopic gestational sac had ruptured. The patient was immediately taken to the operating room for surgical management without obtaining a formal radiology-performed ultrasound. Nonspecific abdominal pain is one of the most common complaints for patients presenting to the ED. The usage of POCUS allows for rapid visualization of the abdominal cavity to diagnose the underlying cause of a patient’s abdominal pain. This case demonstrates that complex etiologies can be reliably visualized and diagnosed without needing to wait for a formal radiology study.

## Introduction

The incidence rate of heterotopic pregnancy is increasing. Though it is still rare, it most commonly occurs in patients undergoing assisted reproductive treatments [1]. Due to the complexity of the condition, and multiple associated diagnostic challenges, patients often present late and suffer from hemorrhaging [2]. Ruptured ectopic pregnancy can be a fatal condition that requires swift diagnosis and intervention.

Hemorrhage caused by a ruptured ectopic pregnancy is the leading cause of pregnancy-related mortality in the first trimester [3,4]. Overall, ruptured ectopic pregnancies account for 2.7% of pregnancy-related deaths [5]. This is one of the conditions in which the usage of point-of-care ultrasound (POCUS) in the emergency department (ED) can help lead to better patient outcomes. The use of POCUS, when compared to radiology-department performed ultrasound (RADUS), leads to significantly shorter mean intervals from ED arrival to ultrasound interpretation, obstetric consult, and transfer to the operating room (OR) arrival for ruptured ectopic pregnancies [6,7].

## Case presentation

A 31-year-old female with a history of infertility presented to the ED with gradually worsening abdominal pain and tenderness in the right lower quadrant which began the previous night following intercourse. The patient had recently undergone infertility treatment, including ovulation induction with clomiphene and a round of intrauterine insemination. The sudden onset of pain was severe enough to elicit crying and vomiting. The use of ice and a heating pad provided minor relief. Rather than presenting to the ED when the pain began, the patient elected to attend a previously scheduled reproductive endocrinology and infertility office appointment. The routine outpatient ultrasound that morning showed a gestational sac in the uterus, a cystic mass in the right adnexa, and fluid in the pelvis. These findings were concerning for heterotopic pregnancy and the patient was instructed to report to the ED by the outpatient OB-GYN physician with suspicion of heterotopic pregnancy.

**Figure 1:**
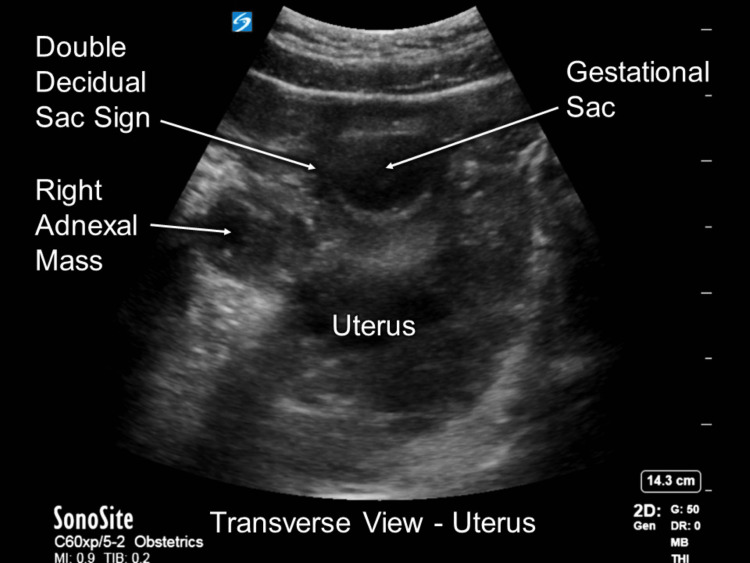
POCUS exam image highlighting the relevant findings. In this view, both the ectopic (right adnexal mass) and intrauterine (gestational sac) pregnancies are visible.

The patient’s initial vital signs were a blood pressure of 114/65 mmHg, heart rate of 109 bpm, respiratory rate of 21 breaths/min, oxygen saturation of 100% on room air, and a temperature of 36.7 ^o^C. Her physical examination found tenderness in the right lower quadrant without guarding or rebounding. The patient’s abdomen was soft and nondistended. There was no vaginal bleeding present. A POCUS FAST examination was performed in the ED and was negative for signs of trauma. Immediately after, a transabdominal POCUS pelvic examination was performed in which the right ovary could be visualized with an ectopic pregnancy and an intrauterine pregnancy (Figure 1).

**Figure 2:**
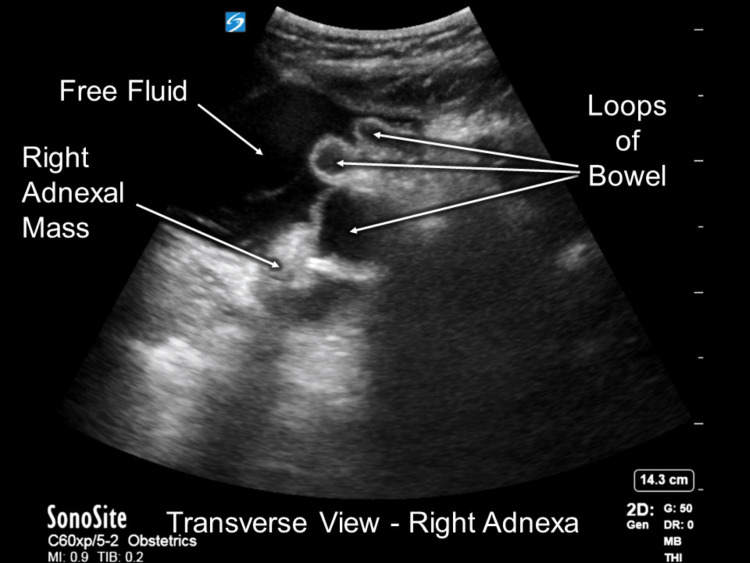
POCUS exam image showing the cystic mass located on the right adnexa and free abdominal fluid. POCUS: point-of-care ultrasound

There was a significant increase in abdominal bleeding noted between the POCUS examination results and the outpatient ultrasound performed an hour prior (Figure 2). These sonographic findings provided sufficient evidence to support the diagnosis of ruptured heterotopic pregnancy.

**Figure 3:**
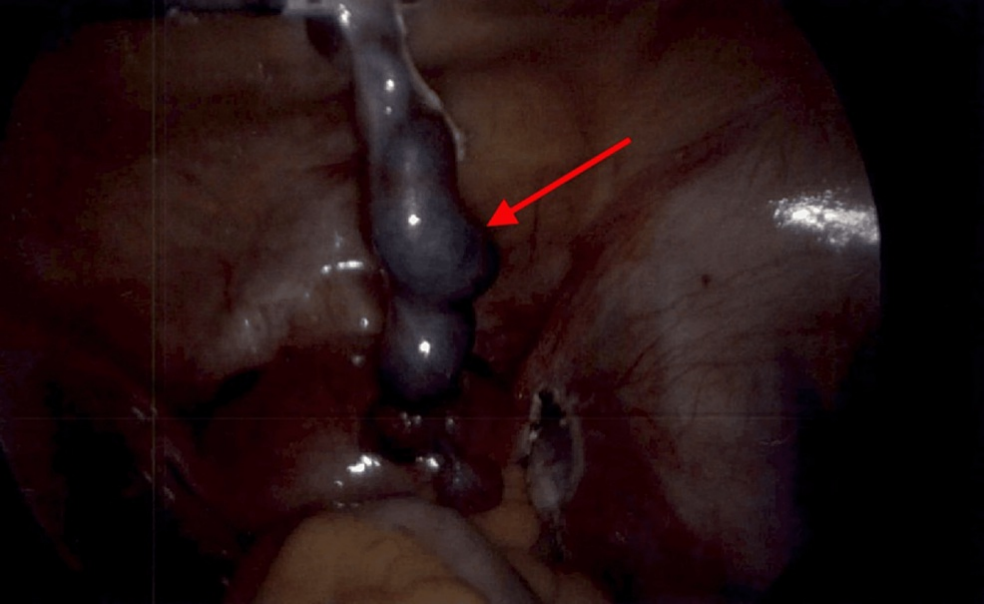
This intraoperative image, taken during the exploratory laparotomy, depicts the ruptured ectopic pregnancy located on the right adnexa.

OB-GYN was consulted in preparation to bring the patient to the OR without formal RADUS; only the POCUS was performed in the ED. The initial diagnosis of heterotopic pregnancy was confirmed during an exploratory laparoscopic procedure. The right fallopian tube had a 1cm paratubal cyst and was dilated due to ectopic pregnancy (Figure 3).

A right salpingectomy was performed along with the evacuation of 100mL of intra-abdominal blood. The operation was performed without complications, and the patient was discharged the next day. Four weeks later, the patient returned to the OR for dilation and curettage via suction of the intrauterine pregnancy, now at six weeks and six days of gestation.

## Discussion

This is not the first utilization of POCUS to diagnose a heterotopic pregnancy in the ED. Current literature of heterotopic pregnancies presenting to the ED have been either misdiagnosed on POCUS or still sent for formal ultrasound evaluation by radiology or OB-GYN [1,2,8,9]. Few cases have been described in which the heterotopic pregnancy is correctly diagnosed via POCUS in the ED and the patient is immediately brought to the OR [10,11]. The ectopic gestational sac had ruptured in only one of the three cases brought to the OR after POCUS [10,11]. It is common for patients with heterotopic pregnancies to undergo in vitro fertilization in the weeks leading up to their diagnosis. All three of the patients’ intrauterine pregnancies were preserved [10,11]. Here we describe a unique case of a patient surgically managed with a ruptured heterotopic pregnancy diagnosed solely via POCUS who eventually required termination of their intrauterine pregnancy weeks later. It is important to note that while the accumulation of free fluid in the abdomen is an indicator of ruptured ectopic pregnancy, it can result from a number of other conditions. Such findings obtained from a POCUS examination should be treated with caution and, when surgical intervention is warranted, an exploratory procedure is recommended to account for the highest number of potential etiologies.

## Conclusions

This case highlights how POCUS can be used to make complex diagnoses in the emergency department without formal or confirmatory ultrasound procedures performed by radiology. Being able to make these diagnoses at the bedside in the ED allows for expedited care, something that is crucial for ruptured ectopic pregnancies and other conditions with a time-dependent prognosis. In this case, using POCUS allowed our team to diagnose and transfer the patient to OB-GYN for surgical management more rapidly than traditional radiology studies could have been performed.
